# Supplementary material for: Healthcare utilization in Canadian children and young adults with asthma during the COVID-19 pandemic
Source: PLoS One. 2023 Jan 13;18(1):e0280362. doi: 10.1371/journal.pone.0280362 (PMC9838850; doi:10.1371/journal.pone.0280362)
Supplement: S1 File — (DOCX) [file pone.0280362.s001.docx]

| Project InitiationThis Section must be Completed Prior to Project Dataset(s) Creation | | | | | | |
| --- | --- | --- | --- | --- | --- | --- |
| **Project Title:** | Health services use among the young Ontario population with asthma and allergic diseases during the COVID-19 pandemic | | | | | |
| **Project TRIM number:** | 2022 0904 152 000 | | | | | |
| **Research Program:** | CDP | | | | | |
| **Site:** | ICES UofT | | | | | |
| **Project Objectives:** | *Insert Project Objectives as listed in the approved ICES Project PIA* | | | | | |
|  | 1. To measure the impact of the COVID-19 pandemic on health services use for children, adults, and pregnant women with asthma and other allergic disease (allergic rhinitis and eczema) in Ontario, Canada 2. To examine the differences of health services use, and delivery outcomes of pregnant women, between the study populations (those asthma, allergic rhinitis and/or eczema) with two control populations (with other chronic illness such as diabetes), and the otherwise healthy general population during the COVID-19 pandemic 3. To examine the changes of patterns of access to health care services in the study populations | | | | | |
| **ICES Project PIA Initial Approval Date:** | *The ICES Employee or agent who is responsible for creating the Project Dataset(s) is responsible for ensuring there is an approved ICES Project PIA and verifying the date of approval prior to creating the Project Dataset(s)* | | | | | |
|  | 2021-Jul-21 | | | | | |
| **Principal Investigator (PI):** | Teresa To | | | | | |
| **Check the applicable box if the PI is an ICES Student/Trainee** | ICES Student  ICES Fellow  ICES Post-Doctoral Trainee  Visiting Scholar | | | | | |
| **Responsible ICES Scientist:** | *Name the Responsible ICES Scientist if the PI is not a Full Status ICES Scientist* | | | | | |
|  |  | | | | | |
| **Project Team Member(s) Responsible for Project Dataset Creation and/or Statistical Analysis and date joined (list all):** | *All person(s) (ICES Analyst, Appointed Analyst, Analytic Epidemiologist, PI, and/or Student) responsible for creating the Project Dataset(s) and/or statistical analysis on the Research Analytics Environment (RAE) and the date they joined the project must be recorded* | | | | | |
|  | Jingqin Zhu Kimball Zhang | | | 2021-Jul-21 2021-Jul-21 | | |
| **Project Team Member(s) who will request RAE folder access (list all):** | *List the project team member responsible for dataset creation who will request access for all members requiring RAE project folder access (e.g. analyst, methodologist, student).* | | | | | |
|  | Jingqin Zhu Kimball Zhang | | 2021-Jul-21 2021-Jul-21 | | |  |
| **Other ICES Project Team Members and date joined (list all):** | *All other Research Project Team Members (e.g., Research Administrative Assistants, Research Assistants, Project Managers, Epidemiologists) and the date they joined the project must be recorded* | | | | | |
|  | Emilie Terebessy | | | 2021-Jul-21 | | |
| **Confirmation that DCP is consistent with Project Objectives:** | *The following individuals must confirm that the ICES Data provided for in this DCP is relevant (e.g., with respect to cohort, timeframe, and variables) and required to achieve the Project Objectives stated in the ICES Project PIA prior to initial Project Dataset creation: 1) PI; 2) Responsible ICES Scientist if the PI is not a Full Status ICES Scientist, or a second ICES Scientist or the Scientific Program Lead if the PI is creating both the DCP and the Project Dataset[s]; 3) ICES Research and Analysis Staff creating the DCP; and 4) ICES Analytic Staff (ICES Employee or agent responsible for creating the Project Dataset[s]). This may be delegated either verbally or via e-mail.* | | | | | |
|  | ***Principal Investigator*** Dr. Teresa To |  | | | 2022-Jul-21 | |
|  | ***Responsible ICES Scientist or Second ICES Scientist/Lead*** |  | | yyyy-mon-dd | | |
|  | ***ICES Research and Analysis Staff Creating the DCP*** Kimball Zhang |  | | yyyy-mon-dd | | |
|  | ***ICES Analytic Staff***  Jingqin Zhu, Kimball Zhang |  | | yyyy-mon-dd | | |
| **Designated ICES Research and Analysis Staff accountable for Project Documentation:** | *The person named (ICES staff) is accountable for ensuring that the approved ICES Project PIA, ICES Project PIA Amendments, and DCP are saved on the T Drive, ensuring ICES Project PIA Amendments are submitted as required, ensuring DCP Amendments are documented, and sharing the final DCP with the PI/Responsible ICES Scientist at project completion* | | | | | |
|  | Jingqin Zhu Kimball Zhang | | | | | |

| **DCP Creation Date and Author:** | *Date DCP was finalized prior to Project Dataset(s) creation* | *Name of person who created the DCP* |
| --- | --- | --- |
|  | ***Date*** | ***Name*** |
|  | 2021- Sept-01 | Kimball Zhang |

| ICES DataThis Section must be Completed Prior to Project Dataset(s) Creation | |
| --- | --- |
| *The ICES Employee or agent who is responsible for creating the Project Dataset(s) must ensure that this list includes only data listed in the ICES Project PIA*  *Changes to this list after initial ICES Project PIA approval require an ICES Project PIA Amendment* | *Mandatory for all datasets that are available by individual year* |
| ***General Use Datasets – Health Services*** | ***Years (where applicable)*** |
| CCRS | April 1, 1996 to Most recent |
| CIHI DAD | April 1, 1996 to Most recent |
| HCD | April 1, 1996 to Most recent |
| NACRS | April 1, 1996 to Most recent |
| NRS | April 1, 1996 to Most recent |
| ODB | April 1, 1996 to Most recent |
| OHIP | April 1, 1996 to Most recent |
| OMHRS | April 1, 1996 to Most recent |
| CIHI SDS | April 1, 1996 to Most recent |
| ERCLAIM | April 1, 1996 to Most recent |
| ***General Use Datasets – Care Providers*** |  |
| IPDB | April 1, 1996 to Most recent |
| ***General Use Datasets – Population*** |  |
| CENSUS | April 1, 1996 to Most recent |
| CONTACT | April 1, 1996 to Most recent |
| POP | April 1, 1996 to Most recent |
| RPDB | April 1, 1996 to Most recent |
| ***General Use Datasets – Coding/Geography*** |  |
| DIN | April 1, 1996 to Most recent |
| REF | April 1, 1996 to Most recent |
| LHIN | April 1, 1996 to Most recent |
| PCCF | April 1, 1996 to Most recent |
| ***General Use Datasets - Facilities*** |  |
| INST | April 1, 1996 to Most recent |
| ***General Use Datasets - Other*** |  |
| ASTHMA | April 1, 1996 to Most recent |
| ODD | April 1, 1996 to Most recent |
| ONMARG | April 1, 1996 to Most recent |
| OLIS | April 1, 1996 to Most recent |
| MOMBABY | April 1, 1996 to Most recent |
| ***Controlled Use Datasets*** |  |
| COVAXON | November 1, 2020 to Most recent |
| OLISC19 | December 1, 2019 to Most recent |
| ***Other Datasets (including PSD and PDC data)*** |  |
| ORGD | April 1, 1996 to Most recent |

| Project Amendments and Reconciliation | | | |
| --- | --- | --- | --- |
| **ICES Project PIA Amendment History (add additional rows as needed):** | *Privacy approval date* | *Person who submitted amendment* | *Note that any changes to the list of ICES Data or Project Objectives require an ICES Project PIA Amendment* |
|  | ***Date*** | ***Name*** | ***Amendment*** |
|  | yyyy-mon-dd |  |  |
| **DCP Amendment History (add additional rows as needed):** | *Date DCP amended* | *Person who made the DCP amendment* | *Note that any DCP amendments involving changes to the list of ICES Data or Project Objectives require an ICES Project PIA Amendment* |
|  | ***Date*** | ***Name*** | ***Amendment*** |
|  | 2021-Oct-19 | Emilie Terebessy | Update project objectives to include pregnant women, add MOMBABY, add covariates involving pregnant women |
| **Date Programs/DCP reconciled** | *The person(s) creating the dataset and/or analyzing the data are responsible for ensuring that the final DCP reflects the final program(s) when the project is completed* | | |
|  | yyyy-mon-dd | | |

| Project Cohort | | |
| --- | --- | --- |
| **Study Design** | Cohort study  Matched cohort study  Case-control study  Cross-sectional study  Other (specify): | |
| **Index Event / Inclusion Criteria**  *(please ensure index event / inclusion criteria are specified with data sources, variables, study period and values or codes)* | Individuals in Ontario  Index Event: Occurance of COVID-19 pandemic on December 1, 2019 | |
| **Estimated Size of Cohort** |  | |
| **Exclusions** *(in order)  (common exclusions are listed in grey italics for consideration)* | *Step* | Description |
|  | 1 | Invalid IKN |
|  | 2 | No Valid Health card number |
|  | 3 | No valid Ontario residence code |
|  | 4 | No valid age value at ICES |
|  | 5 | No valid gender |
|  | 6 | Death date prior to index date |
|  | 7 | Age over 60 |

| Project Time Frame Definitions | | |
| --- | --- | --- |
| Look-back Window  Observation Window  (in which to look for outcomes)  **Index Event Date**  Accrual Window  Max Follow-up Date | |  |
| **Accrual Start/End Dates** | April 1, 1996 to Most recent |  |
| **Max Follow-up Date** | Most recent |  |
| **When does observation window terminate?** | Max Follow-up date (Most recent) |  |
| **Lookback Window(s)**  *(please ensure lookback windows are defined with start and end dates and in relation to the index event date)* | 1 year |  |

| Variable Definitions (add additional rows as needed) *A few key points to keep in mind:*   1. *Please ensure codes, data sources, diagnosis types and lookback periods (if applicable) are provided for all definitions listed below and that codes are provided in Excel format. If borrowing codes from another project, please list all the codes here* 2. *There are maximum number of digits that can be specified using ICES data (ICD 9 CA codes are up to maximum of 4 digits, ICD 10 CA codes are 6 digits, OHIP diagnosis codes are 3 digits)* | | |
| --- | --- | --- |
| **Main Exposure or Risk Factor** | COVID-19 Pandemic |  |
| **Primary Outcome Definition** | 1. Asthma incidence as identified by the following validated administrative definition (Gershon et al. 2009, To et al. 2006): ≥ 1 asthma hospital admission; or ≥ 2 asthma OHIP claims in 2 consecutive years 2. Allergic rhinitis as identified by its code from the International Statistical Classification of Diseases and Related Health Problems, Tenth Revision, Canada (ICD-9: 477 and ICD-10: J301–J304) 3. Eczema as identified by its code from the ICD-10-CA (ICD-9: 691.8 and ICD-10: L20) 4. Rates of health services use (hospitalizations, ED visits, physician office visits, telemedicine) 5. Costs of health services use (hospitalizations, ED visits, physician office visits, telemedicine) 6. Rates of health outcomes (e.g., mortality, laboratory tests, prescription drug utilization) 7. COVID-19 vaccination rates (partial and full) 8. Distance travelled in seeking health care 9. Delivery outcomes of pregnant women from the ICD-10-CA e.g. outcome of delivery (ICD-9: V27 and ICD-10: Z37) and caesarean section (ICD-9/CCP: 86 and ICD-10/CCI: 5MD60) |  |
| **Secondary Outcome Definition(s)** |  |  |
| **Baseline Characteristics** | N/A |  |
| **Other Variables** | Age, sex, location of residence, rural/urban status, institutions/hospitals (e.g. teaching hospital, community hospitals), vaccination status, and socioeconomic status (income quintile, Ontario Marginalization Index: indices of material deprivation, ethnicity, dependence and residential instability), maternal conditions and pregnancy outcomes (e.g. pre-eclampsia, pregnancy induced hypertension, prematurity) |  |

| Analysis Plan and Dummy Tables (expand/modify as needed) *(please ensure the analysis plan is outlined with dummy tables (can be a separate document)*  *and clear specification of exposures / outcomes / covariates for each model)* | | |
| --- | --- | --- |
| **Descriptive Tables (insert or append dummy tables), e.g.:** | | |
| **Table 1. Baseline characteristics according to primary/secondary exposure** | | |
| **Table 2. Outcomes according to primary/secondary exposure** | | |
| **Table 3. Covariates (baseline characteristics) according to outcomes** | | |
| **Statistical Model(s)** | | |
| **Type of model** | Poisson regression | |
| **Primary independent variable** | COVID-19 Pandemic | |
| **Dependent variable** | 1. Rate of health service use 2. Cost of health service use | |
| **Covariates** | Age, sex, location of residence, rural/urban status, institutions/hospitals, vaccination status, socioeconomic status (income quintile, Ontario Marginalization Index: indices of material deprivation, ethnicity, dependence and residential instability), maternal conditions and pregnancy outcomes (e.g. pre-eclampsia, pregnancy induced hypertension, prematurity) | |
| **Statistical Model(s)** | | |
| **Type of model** | Linear regression | |
| **Primary independent variable** | COVID-19 Pandemic | |
| **Dependent variable** | 1. Cost of health service use 2. Distance travelled | |
| **Covariates** | Age, sex, location of residence, rural/urban status, institutions/hospitals, vaccination status, socioeconomic status (income quintile, Ontario Marginalization Index: indices of material deprivation, ethnicity, dependence and residential instability), maternal conditions and pregnancy outcomes (e.g. pre-eclampsia, pregnancy induced hypertension, prematurity) | |
| **Sensitivity Analyses** |  | |
| **Type of model** |  | |
| **Primary independent variable** |  | |
| **Dependent variable** |  | |
| **Covariates** |  | |
|  | |  |

| Quality Assurance Activities | | | |
| --- | --- | --- | --- |
| **RAE Directory of SAS Programs** |  | | |
| **RAE Directory of Final Dataset(s)** | *The* *final analytic dataset for each cohort includes all the data required to create the baseline tables and run all the models. It should include all covariates for all models such as patient risk factors, hospital characteristics, physician characteristics, exposure measures (continuous, categorical) and outcomes. It should include covariates that were considered but didn’t make the final cut. This would permit an analyst to easily re-run the models in the future.* | | |
|  |  | | |
| **RAE README file available:** Yes No | | | |
| **Date results of quality assurance tools for final dataset shared with project team (where applicable):** | | |  |
|  | | **%assign** | yyyy-mon-dd |
|  | | **%evolution** | yyyy-mon-dd |
|  | | **%dinexplore** | yyyy-mon-dd |
|  | | **%track / %exclude** | yyyy-mon-dd |
|  | | **%codebook** | yyyy-mon-dd |
| **Additional comments:** | |  | |
