# Supplementary figures and images for: Healthcare utilization in Canadian children and young adults with asthma during the COVID-19 pandemic
Source: PLoS One. 2023 Jan 13;18(1):e0280362. doi: 10.1371/journal.pone.0280362 (PMC9838850; doi:10.1371/journal.pone.0280362)

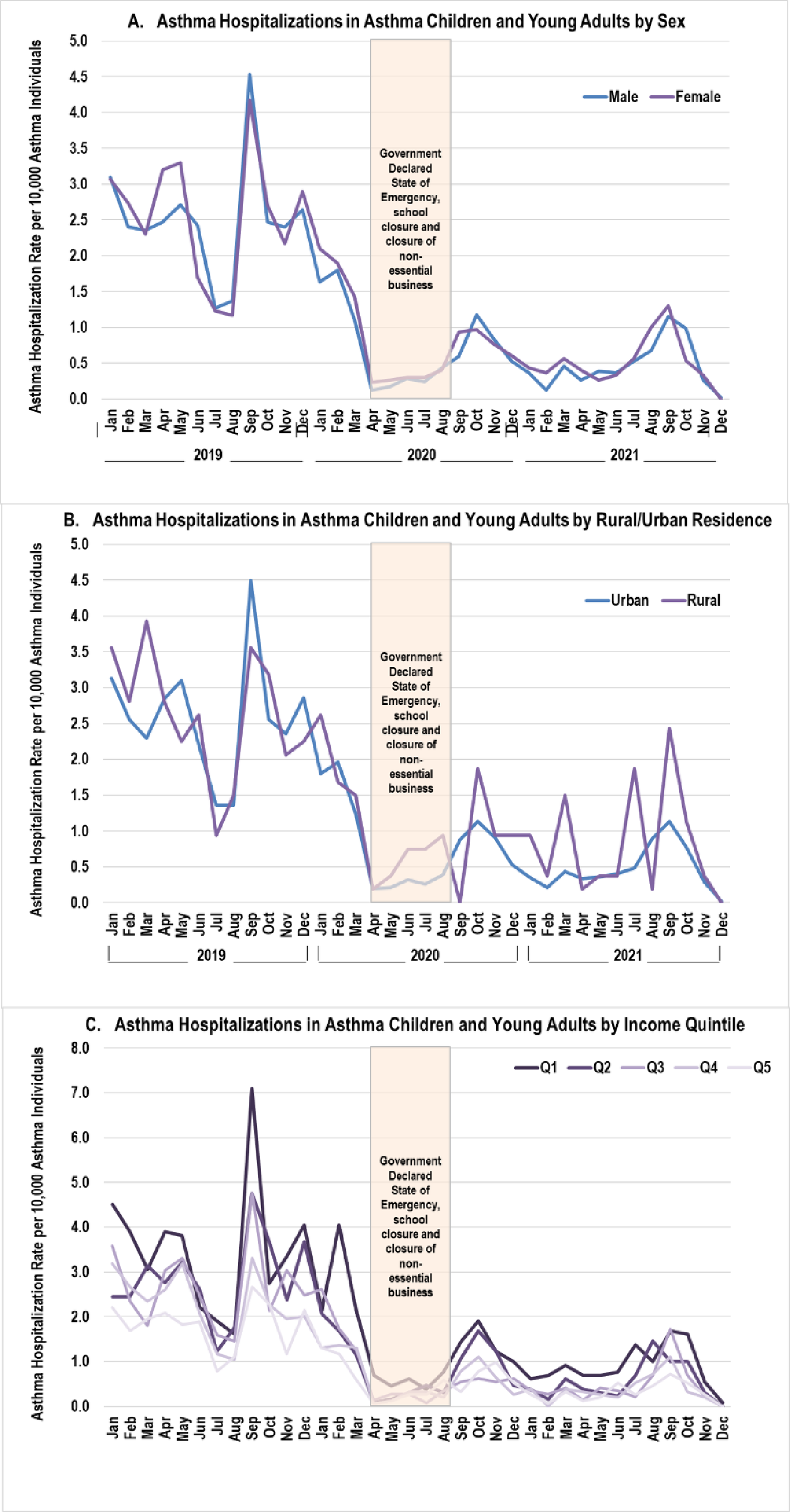

Supplement: S1 Fig — (TIF) [file pone.0280362.s002.tif]

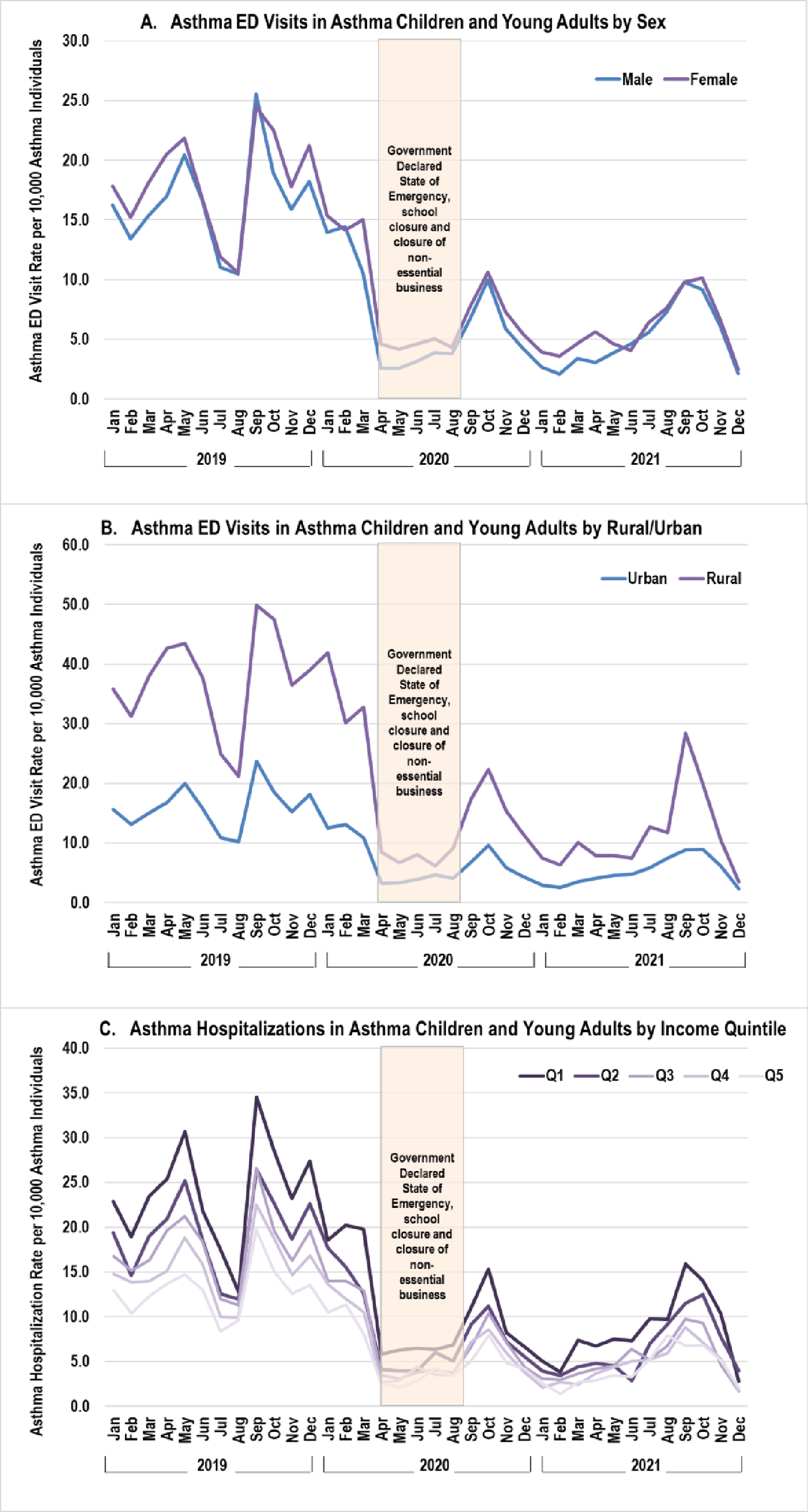

Supplement: S2 Fig — (TIF) [file pone.0280362.s003.tif]

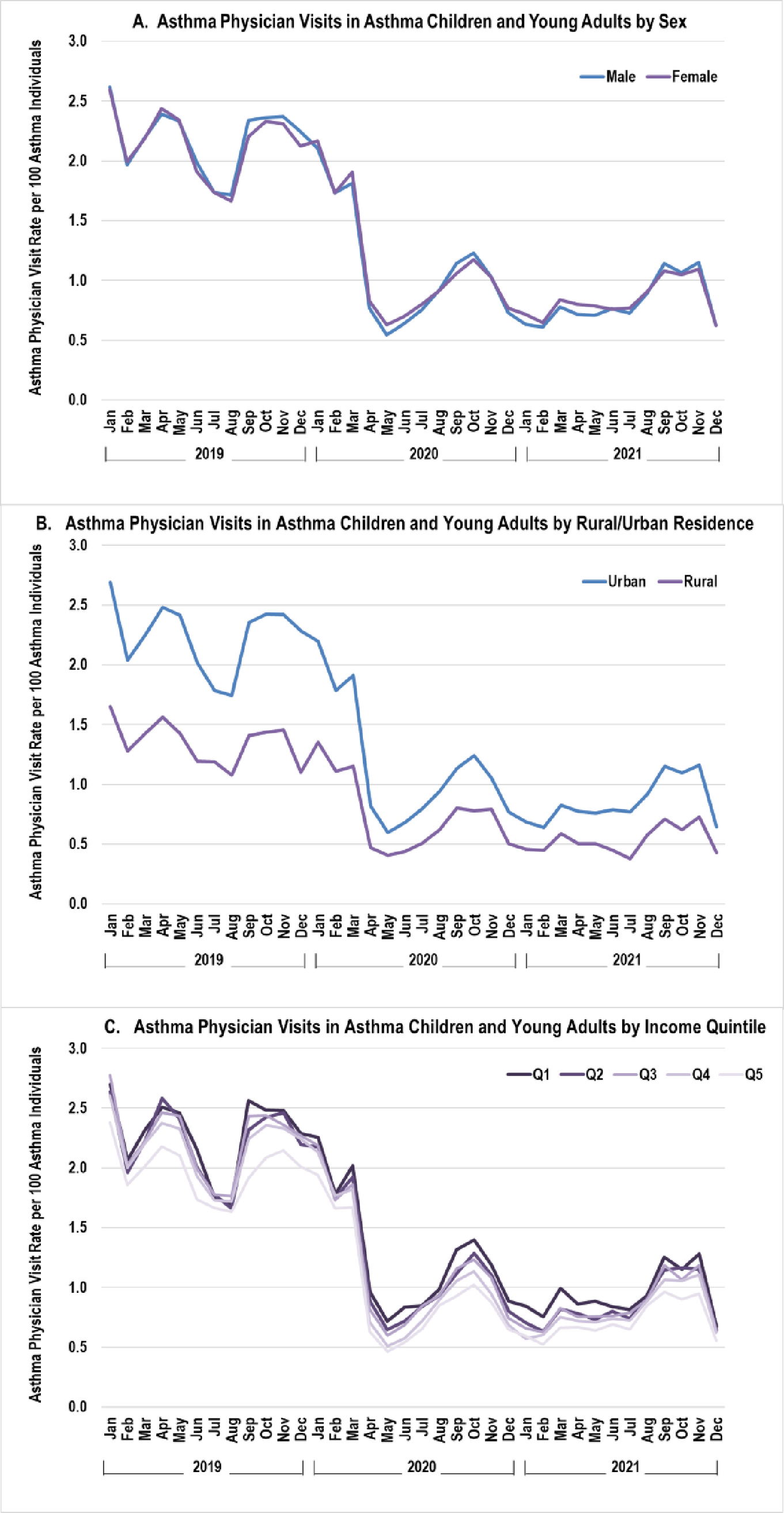

Supplement: S3 Fig — (TIF) [file pone.0280362.s004.tif]

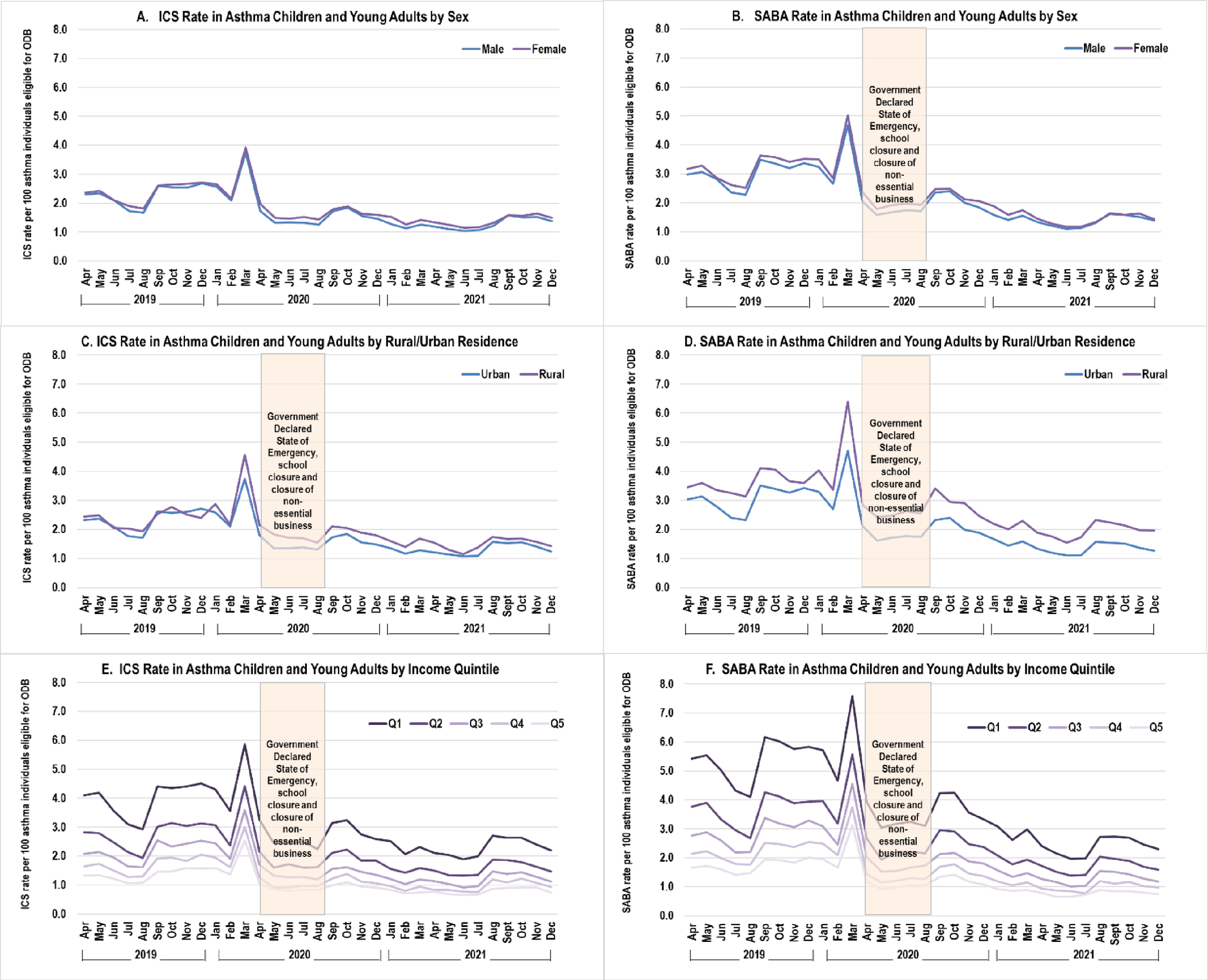

Supplement: S4 Fig — (TIF) [file pone.0280362.s005.tif]
