# Supplementary material for: Healthcare utilization in Canadian children and young adults with asthma during the COVID-19 pandemic
Source: PLoS One. 2023 Jan 13;18(1):e0280362. doi: 10.1371/journal.pone.0280362 (PMC9838850; doi:10.1371/journal.pone.0280362)
Supplement: Attachment — Submitted filename: PONE-D-22-16786_ResponsetoReviewers_2022-10-18_CC.docx [file pone.0280362.s006.docx]

**RESPONSE TO REVIEWERS’ COMMENTS**

**Healthcare Utilization in Canadian Children and Young Adults with Asthma**

**during the COVID-19 Pandemic**
PONE-D-22-16786

*Please note that all line and page numbers cited correspond to the clean version of the revised manuscript.*

# COMMENTS FROM REVIEWER #1

**Well written paper. No incredible findings but the conclusion doesn't attempt to overstate the possible causes. Overall I have no concerns about the papers research methodology or findings. No spelling mistakes so that is great.**

No comments to respond to.

# COMMENTS FROM REVIEWER #2

**The authors use the national health administrative database for collecting data on healthcare use: disease burden (number of hospitalizations, ED, outpatient visits) and treatment prescription in the asthmatic population between 0-25 years. The use of this database gives the opportunity to study a large sample size and to provide longitudinal data as highlighted in the discussion of this manuscript. However, some concerns remain unresolved:**

1. **The diagnosis of asthma is defined by the authors based on a low threshold (>or=1 hospitalization for asthma or >or=2 outpatient visits for asthma in 2 consecutive years). Especially in the lowest age category this could include an important selection bias. Children from 0-4y can have sporadic viral induced wheezing without underlying asthma as for example during RSV infection, necessitating hospitalization. This concern (of mixing ‘sporadic viral induced wheezing’ with asthma) has not been discussed in the manuscript and should at least earn some clarification in the discussion. An important amount of children who are in the age category 0-4 years prior to the pandemic are expected to outgrow this problem 2 years later. Besides, including the youngest age category increases the average monthly rate of all the outcomes measured, illustrated in Figure 1. Subanalysis excluding this age-population would add valuable information.**

We included children 0-4 years in our study for the following reasons:

- We agree that confirming diagnosis of asthma in children younger than 5 years is challenging for reasons that the reviewer indicated. However, the case definition we used in this study was previously validated including young children (see reference in below). In those aged 0-2 and 3-5, the sensitivity was 75.0%, 96.0% and specificity was 83.9% and 84.2% respectively. With these sensitivity and specificity estimates, we are comfortable in including these young children with asthma in the study.
- The estimates provided by the OASIS (Ontario Asthma Surveillance Information System: <https://lab.research.sickkids.ca/oasis/data-tables/>) showed asthma-specific health care utilization in young children remained high and higher than older children. It is therefore especially important to monitor and compare how the pandemic may have further impacted these young children.
- Our study population included all Ontario residents aged ≤25 years old with prevalent asthma in the period of January 1, 2019 to December 31, 2021. While those who were 0-4 in 2019 would have been 2 years older in 2021, since this is a population-based repeated cross-sectional study, children 0-4 with prevalent asthma in each year were included.
- Lastly, all our analyses were stratified by age groups, the rates and rate ratios reported were age-specific.

*Reference*: To, T., Dell, S., Dick, P. T., Cicutto, L., Harris, J. K., MacLusky, I. B., & Tassoudji, M. (2006). Case verification of children with asthma in Ontario. *Pediatric Allergy and Immunology*, 17(1), 69–76. https://doi.org/10.1111/j.1399-3038.2005.00346.x

2. **Study population is defined as children and young adults. In the introduction the authors describe that the target population is children and youth. Youth is defined by WHO by individuals with age between 15 and 24 years old. Young adults are defined between 12y and 30 years. Why do authors choose the population until 25 years included and not until 24years? Has this been arbitrary chosen? Please clarify and adapt in the analysis if feasible.**

We chose a population up to 25 years due to public drug coverage availability among those ≤25 years in Ontario via the OHIP Plus Ontario Drug Benefit (ODB) program. Prescriptions filled by this population are captured in the Ontario Drug Benefit database. We clarified this in the “Study Design & Population” section on page 3; an updated analysis is not required for this.

3. **Rephrase the sentence, which sounds odd: P4 prescriptions on asthma medications filled and covered by OHIP were captured through the ODB database were identified…**

We rephrased the sentence on page 4: “Asthma medication prescriptions filled, as covered by OHIP Plus, were captured through the ODB database. These prescriptions were identified using the Drug Identification Number.”

4. **In the discussion part P18: rate of ICS increased early in the pandemic, but also SABA**

We expanded the “Discussion” section on page 18 to provide a brief explanation on the increased rate of SABA prescriptions, while citing additional literature: “While we also observed a similar spike involving SABA, there are limited literature quantifying asthma rescue medication use early in the COVID-19 pandemic. Instead, most literature on asthma rescue medication like SABA involved longer COVID-19 pandemic periods with little comparisons on usage between the early and later parts of the pandemic. These authors found an overall decreased use of SABA during COVID-19.”
